# Effect of health seeking behaviour of caregivers on severe malaria outcome in under-fives seen in a tertiary health institution in Nigeria

**DOI:** 10.4314/gmj.v54i3.6

**Published:** 2020-09

**Authors:** Damian U Nwaneri, Ayebo E Sadoh

**Affiliations:** Department of Child Health, University Of Benin Teaching Hospital, P.M.B. 1111, Benin City, Nigeria)

**Keywords:** behaviour, caregivers, severe malaria, health-seeking, outcome

## Abstract

**Background:**

Recognition of the symptoms and seeking prompt treatment in a health facility is a major means of reducing morbidity and prevention of mortality from severe malaria in under-fives.

**Objectives:**

To document the effect of health-seeking behaviour of caregivers and severe malaria outcome in underfives seen in a tertiary health institution in Nigeria.

**Design:**

A descriptive cross-sectional study carried out from July 2012 – June 2013. Data were obtained using a researcher-administered questionnaire.

**Subjects:**

Caregivers and children (6 – 59 months) who presented with features of severe malaria according to World Health Organization criteria.

**Results:**

Of the 120 caregivers mean [SD] age (31.4 [7.0] years) /child pairs (24 [14.7] months), 35 (29%) caregivers had appropriate health-seeking behaviour. The commonest place visited for initial healthcare before presentation was the patent medicine vendors by 87 (73%) caregivers. Seventy-seven per cent of caregivers who did not have appropriate health-seeking behaviour were from the lower family social class (*p* = 0.03). Caregivers whose children presented with severe anaemia were significantly more likely to have appropriate health-seeking behaviour (*p* =0.00). The mortality rate of severe malaria was 15 per 1000; of which 94% were children whose caregivers did not have appropriate health-seeking behaviour. Age younger than 2 years (*p* = 0.02), cerebral malaria (*p* = 0.01) and jaundice (*p* = 0.03) significantly predicted mortality in the children irrespective of the caregivers' health-seeking behaviour status.

**Conclusion:**

Less than a third of the caregivers had appropriate health-seeking behaviour for their under-fives with severe malaria, and the majority of these were from the lower family social class. Cerebral malaria and jaundice significantly predicted mortality in children with severe malaria irrespective of caregivers' health-seeking behaviour status.

**Funding:**

The study was self-sponsored by the authors

## Introduction

Severe malaria contributes significantly to paediatric emergency room admissions in sub-Saharan Africa.^1^ Currently, 545,000 people died from malaria in 2017.^2^ It has severe consequences in under-fives as death usually occurs within the first 2 – 3 days of onset of illness and the worst outcome is within the first 3 years of life.^3^ Common causes of death from severe malaria include severe malarial anaemia, hypoglycaemia, cerebral malaria, acidosis and hypovolemic shock.^4^–^7^ Recent hospital-based study in Benin City Nigeria showed that the prevalence rate of severe malaria is 38.3% with a mortality rate of 60 per 1000.^8^ In Nigeria, the National Malaria Elimination Program (NMEP) has instituted some strategic plans to educate caregivers through a comprehensive malaria control programme from the Federal to the communities at the Local Government level.^10^ These programmes included long-lasting insecticide-treated nets (LLIN) campaign, training and re-training of healthcare providers on case management of malaria, and step-down Advocacy Communication and Social Mobilization (ACSM) strategy for malaria control at the community level. A drastic reduction in the prevalence of severe malaria is expected to occur following implementation of these control/ interventional programmes The actual outcome of this control strategy, however, may largely depend on health-seeking behaviour of the primary caregivers of these children.

Malaria disease usually starts as a simple acute febrile illness and if left untreated, progresses to the severe (or complicated) disease usually within 72 hours with predicted high mortality.^3^–^5^ Some studies in Nigeria and other malaria endemic regions have shown that majority of households initially visit the patent medicine vendors (PMVs) and other sources for treatment (including neighbours) for simple acute malaria, before finally attending a health facility for proper management.^8^,^11^–^13^ This finding could be attributed to the close-to-client operation of the PMVs (being available virtually in every community and their operation is as close as possible to the homes of patients/ caregivers). Severe malaria, on the other hand, is a medical emergency and requires prompt evaluation and treatment in a well-organised health facility. In under-fives, a delay in accessing treatment for malaria beyond 48 hours from the onset of symptoms usually progresses to severe morbidity and increases fatality.^8^

To reduce the malaria burden, the World Health Organization (WHO) recommends home treatment of uncomplicated malaria with the recommended antimalarial drugs within 24 hours of the onset of symptoms or seeking for prompt treatment in a health facility within 24 hours of onset severe malaria symptoms.^1^,^2^ The management of severe malaria remains challenging in most malaria endemic regions. This is largely because management outcome depends mainly on early (within 24 hours of the onset of symptoms) presentation of the children to a health facility as well as the presence of the expert/ supportive facilities.^3^,^8^ Thus, appropriate health-seeking behaviour (HSB) by caregivers is expected to reduce severe malaria mortality.

However, some factors such as poverty, distance to health facilities, cost of transportation, and increased waiting time in hospitals had been documented as being responsible for seeking for health care elsewhere than the health facility.^14^–^16^ Agu *et al*^14^ outlined that lack of money was the commonest reason for not seeking treatment first in a health facility. Jimoh *et al*^15^observed that it costs USD7.41 per person in a household in Nigeria for full course treatment of uncomplicated malaria with the recommended Artemisinin-based combination therapy (ACT).

This cost was not within reach of most families in Nigeria for the treatment of uncomplicated malaria. Nwaneri *et al*^8^ had documented that the close-to-client operation (being available virtually in every community and their operation is as close as possible to the homes of patients/ caregivers) as one of the major factors to high patronage of the PMVs by caregivers. Despite these documentations, most of these studies did not seek for any association between HSB and malaria outcome. This present study, therefore, sought the relationship between the HSB of caregivers and mortality from severe malaria in under-5s and to document relationship between HSB and socio-demographic factors.

## Methods

This study was carried out at the Children's Emergency Room (CHER) of University of Benin Teaching Hospital (UBTH), Benin City, South-south Region of Nigeria. UBTH is a 750 bedded tertiary health facility that serves the people of Edo State and neighbouring states of Delta in the south-south; Ondo (south-west), Kogi (north-central) and some parts of Anambra States in the southeast region. Majority of these States are within the tropical rain forest where malaria transmission is holoendemic and stable throughout the year, especially in under-fives.^17^ The CHER of the hospital has a total bed capacity of 15 and an average of three cases of severe malaria per week.

### Sample size calculation

This was a cross-sectional descriptive study and the study was carried out from June 2012 to July 2013. A sample size 110 was determined based on 95.0% confidence interval and statistical power of 80.0% using the formula described by Araoye (2007).^18^ However, to accommodate attrition of 10.0%, a total of 120 caregivers and their well-nourished 120 children (aged 6 – 59 months) with confirmed malaria following standard protocols described by Cheesebrough^20^ were recruited in the study.

The children presented with one or more features of severe malaria, according to the World Health Organization (WHO) criteria.^1^ Excluded from this study were children with clinical/laboratory evidence of foci of other infections and malnutrition.^21^ Malnutrition was defined as stunting wasting and under-weight for children whose height for age Z-score, weight for height Z-score and weight for age Z-score were below -2 standard deviation of the reference median value.^22^

The dependent variables in this study were appropriate HSB which was defined for this study as the presentation to a health facility within 24 hours of the onset of features of severe malaria, and inappropriate HSB is when a child with features of severe malaria presented after 24 hours of onset symptoms.^1^,^9^ This definition was deduced from the documentation of Greenwood *et* al^3^ that death from severe malaria is usually within 48 hours of the onset of symptoms.

The independent variables were the ages of the children, their gender, family social class, household size and ages and level of education of caregivers. The family social class was determined using the method described by Olusanya *et al.*^23^ The outcome variable sought for in this study included severe malaria morbidity (the severe malaria features at presentation defined by the WHO criteria) and mortality.^1^ Mortality was defined as death within 48 hours of presentation in the health facility attributable to morbidities of severe malaria.^3^

### Data collection and sampling technique

At recruitment, a semi-structured interviewer administered questionnaire was used to obtain relevant data from each child's caregiver after obtaining written informed consent. The questionnaire was validated by an extensive literature review and was pre-tested on ten caregivers who were excluded from the final analysis. Each caregiver/child pair was assigned an identification number which was used for the questionnaire and laboratory samples. The questionnaire sought information on the child's and caregivers' demographic characteristics, information on causes and symptoms of severe malaria and what was done for the child at the onset of illness. Each child was examined clinically, and all features of severe malaria were documented.

### Ethical considerations

The ethical certificate for this study was obtained from the Research and Ethics Committee of UBTH Benin City Protocol Number ADM/E22/A/vol.VII/741. Written informed consent was obtained from each caregiver.

### Data analysis

The data obtained in this study were recorded in a Microsoft Excel 2007 sheet and the analysis was done using the statistical package for scientific solutions (SPSS) version 16.0 (Chicago, Illinois, USA). Further analysis was done using GraphPad InStat software (GraphPad Software Inc, San Diego 92130, USA) where applicable. Quantitative variables (such as the age of the children, number of days of illness before presentation, and malaria parasite count) were summarised using means and standard deviations where appropriate at the 95^th^ per cent confidence level. Frequency tables and charts were constructed as appropriate. The significance of associations was tested using chi-square and Fisher's exact test as appropriate. Logistic regression model showing socio-demographic and severe malaria features of study participants predicting mortality irrespective of the caregivers HSB status of the caregivers was carried out. The level of significance of each test was set at *p* < 0.05.

## Results

Of the 120 children recruited in the study; their mean [SD] age was 24.0 [14.7] months and that of their caregivers was 31.4 [7.0] years. There were 71 (59%) male children and 49 (41%) females. Socio-demographic characteristics of the caregivers/ children are shown in Table 1. Majority of the children 43 (36%) in this study were aged 12 – 23 months and half of the study participants were from the lower family social class.

**Table 1:** Characteristics of the study participants

Characteristics	N (%)
**CHILDREN**	
***Gender***	
**Male**	71 (59)
**Female**	49 (41)
***Age group (months)***	
**< 12**	29 (24)
**12 – 23**	43 (36)
**24 – 35**	29 (24)
**36 – 47**	13 (11)
**48 – 59**	6 (5)
***Family social class***	
**Upper**	23 (19)
**Middle**	36(30.0)
**Lower**	61(51.0)
***Household size***	
**Small (≤ 5)**	111 (93)
**Large (≥ 6)**	9 (7)
**CAREGIVER**	
***Age group of caregivers (years)***	
**16 – 25**	49 (41)
**26 – 35**	59 (49)
**36 – 45**	9 (8)
**> 45**	3 (2)
***Level of education of caregivers/ mothers***	
**Tertiary**	23 (19)
**Secondary**	45 (38)
**Primary**	45 (38)
**No formal**	7 (5)

**Figure 1:**
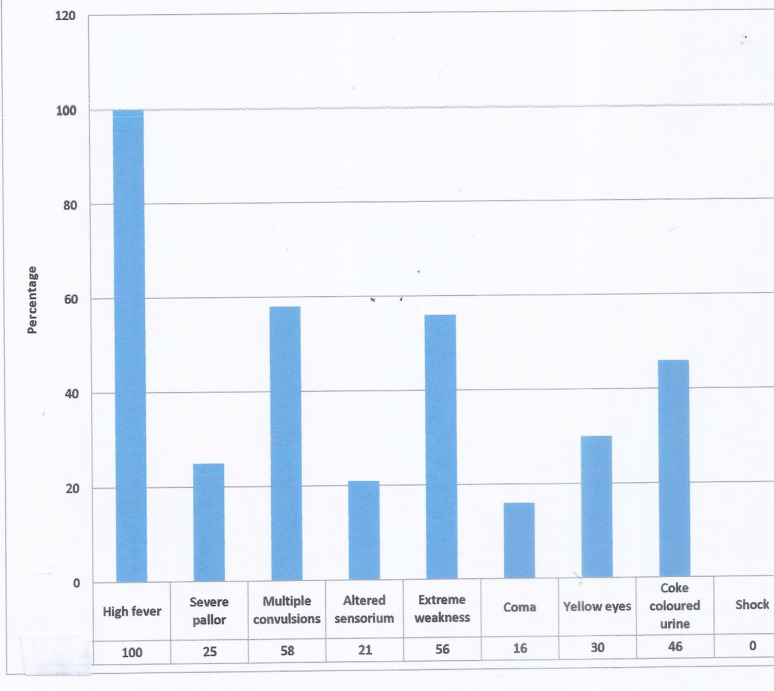
Symptoms of severe malaria as volunteered by the caregivers There were multiple responses and features at presentation*

Concerning causes of malaria, 95% of caregivers recognised mosquitoes as the cause of malaria while the remaining 5% attributed malaria to the dirty environment, bad water and witchcraft. Figure 1 is a bar chart showing the symptoms of severe malaria as volunteered by the caregivers.

**Figure 2:**
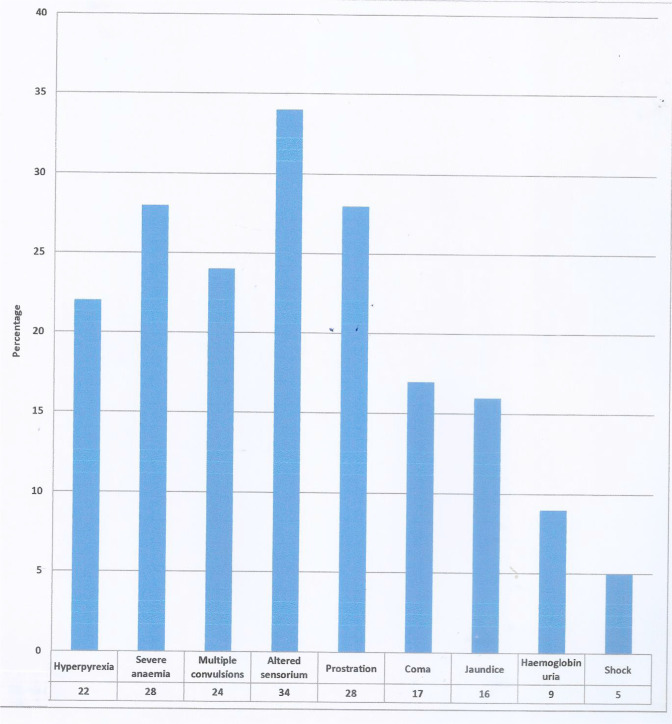
Features of severe malaria identified by the physician There were multiple responses and features at presentation*

All the children were said to have presented with high fever; 70 (58%) had multiple convulsions, 67 (56%) and 55 (46%) had coke coloured urine. Figure 2 shows a bar chart of features of severe malaria identified by the physician at presentation. The most common features identified by the physician was altered sensorium 41 (34%); then multiple convulsions 29 (24%); severe anaemia and prostration 33 (28%) respectively. There were multiple presenting features.

Mean [SD] duration of illness before presentation was 4.2 [3.5] days and the mean [SD] temperature of the children at presentation was 38.0 °C [1.2] °C. The mean [SD] packed cell volume at presentation was 22.7% [9.6] %; and the Mean [SD] malaria parasite count of the children was 3069.0 [2218.0] parasite per microlitre. Hyper-parasitaemia was not observed in any of the children.

Caregivers who had appropriate health-seeking behaviour (HSB) were 35 (29%) while 85 (71%) had inappropriate HSB. The commonest place for initial seeking for health care by the caregivers was the patent medicine vendor by 87 (73%) caregivers, neighbours/ traditional doctors 17 (14%) and health facilities namely private facilities/ health centres 16 (13%).

Table 2 shows the association between appropriate HSB by the caregivers and some socio-demographic characteristics of the study participants. Most caregivers who did not have appropriate HSB were significantly from the lower social class (χ^2^ = 7.34, df = 2, *p* = 0.03).

**Table 2:** Relationship between socio-demographic characteristics of the study participants and healthseeking behaviour by the caregivers

Socio-demographic characteristics	Appropriate HSB			
CHILDREN	Yes	No	χ^2^	p-value
***Age Group (Months)***				
**< 12 (n = 29)**	4 (14)	25(86)		
**12 – 23 (n = 43)**	15 (35)	28(65)	5.40	0.25
**24 – 35 (n = 29)**	10 (35)	19 (65)		
**36 – 47** (n = 13)	5 (39)	8 (61)		
**48 – 59 (n = 6)**	1 (17)	5 (83)		

***Family social class***				
**Upper (n = 23)**	12 (52)	11 (48)		
**Middle (n = 36)**	9 (25)	27 (75)	7.34	0.03*
**Lower (n = 61)**	14 (23)	47 (77)		

***Household size***				
**Small (n = 111)**	34 (31)	77 (69)	1.54	0.22
**Large (n = 9)**	1 (11)	8 (89)		

**CAREGIVER**				
***Age group (Years)***				
**16 – 25 (n = 20)**	7 (35)	13 (65)		
**26 – 35 (n = 73)**	24 (33)	49 (67)	3.87	0.28
**36 – 45 (n = 24)**	4 (17)	20 (83)		
**> 45 (n = 3)**	0 (0.0)	3 (100)		

***Level of education***				
**Tertiary (n = 23)**	11 (48)	12 (52)		
**Secondary (n = 45)**	12 (27)	33 (73)	5.06	0.17
**Primary (n =45)**	10 (22)	35 (78)		
**No formal (n = 7)**	2 (29)	5 (71)		

HSB = Health Seeking Behaviour

Appropriate HSB did not significantly depend on the educational status (χ^2^ = 5.06, df = 2, *p* = 0.17) and ages (χ^2^ = 3.87, df = 2, *p* = 0.28) of the caregivers. It was also not significantly depend on ages of the children (χ^2^ =5.40, df = 2, *p* = 0.25) and the household size (χ^2^ = 1.54, df = 2, *p* = 0.22).

Table 3 shows the association of health-seeking behaviour and features of severe malaria at presentation (severe malaria morbidity). Children whose caregivers did not have appropriate HSB significantly presented with severe anaemia (*p* = 0.00). HSB of the caregivers did not significantly affect the other features of severe malaria presentation of the children.

**Table 3:** The effect of health-seeking behaviour on features of severe malaria at presentation (severe malaria morbidity)

	Appropriate HSB				
	Yes (%) n = 35	No (%) n=85	χ^2^	OR	*p*- value
**Severe malaria** **features** **(morbidity)**					
***Multiple*** ***Convulsions***					
**Yes**	25 (71)	45 (53)	3.49	2.22	0.06
**No**	10 (29)	40 (47)			
***Cerebral malaria***					
**Yes**	10 (29)	31 (36)	0.69	0.70	0.41
**No**	25 (71)	54 (64			
***Severe anaemia***					
**Yes**	4 (11)	32 (38)	+	0.21	0.00*
**No**	31 (89)	53 (62			
***Hyperpyrexia***					
**Yes**	7 (20)	20 (24)	0.91	0.83	0.91
**No**	21 (80)	50 (76)			
**Missing data**	22				
***Prostration***					
**Yes**	7 (20	26 (31)	1.39	0.60	0.24
**No**	28 (80)	59 (69			
***Haemoglobinuria***					
**Yes**	4 (11)	7 (8)	+	1.4	0.58
**No**	31 (89)	78 (92)			
***Jaundice***					
**Yes**	3 (9)	12 (14)	+	0.6	0.40
**No**	32 (91)	73 (86)			
***Shock***					
**Yes**	0 (0)	6 (7)	+	0.20	0.11
**No**	35 (100)	79 (93)		2.22	

+Fisher's Exact; HSB = Health Seeking Behaviour

Mortality rate observed in this study was 150 per 1000; of which 94% were children whose caregivers did not have appropriate HSB (χ^2^ = 5.71, OR = 8.5, *p* = 0.02). These children were 8 and half times more likely to die from severe malaria,

Table 4 shows the final step of the logistic regression model showing severe malaria and socio-demographic features of the study participants predicting mortality (independent variable) in the children irrespective of their caregivers HSB (constant variable among all the children). Cerebral malaria and jaundice were significant predictors of mortality in the children. Presence of cerebral malaria features such as coma and altered sensorium in a child predicted 1.7 times the likelihood of death (β = -1.80, OR = 0.17, *p* = 0.02); likewise jaundice by 1.2 times (β = -2.15, OR = 0.12, *p* = 0.03). Age of the children was the only socio-demographic feature that significantly predicted mortality in the children. The younger the child, the four times likelihood of death from severe malaria (β = 1.43, OR = 4.2, *p* = 0.02).

**Table 4:** The final step of the logistic regression model showing socio-demography and severe malaria features of the study participants predicting mortality (independent variable) in the children irrespective of their caregivers HSB status (constant variable among all the children)

Socio-demography	β	OR	95%CI	*p*-value
**Age Group**				
**(<24 Vs ≥24 months)**	1.43	4.2	1.22, 14.42	0.02*
**Family social class**	-0.89	0.40	0.09, 1.81	0.24
**Household Size**	-0.17	0.85	0.59, 1.23	0.37
**Age of the caregivers**	-0.00	1.00	0.91, 1.10	0.98
**Caregiver's level of** **education**	-0.12	0.90	0.26, 2.98	0.84

**Features of**	**β**	**OR**	**95%CI**	
**severe malaria**				
**Multiple convulsions**	-0.97	0.40	0.09, 1.66	0.20
**Cerebral malaria**	-1.80	0.17	0.05, 0.64	0.01*
**Severe anaemia**	-0.08	0.93	0.11, 1.50	0.91
**Hyperpyrexia**	-0.21	0.81	0.20, 3.24	0.77
**Prostrations**	0.39	1.50	0.32, 6.76	0.61
**Haemoglobinuria**	19.61	3.30	0.00, --	1.00
**Jaundice**	-2.15	0.12	0.02, 0.80	0.03*
**Shock**	-1.66	0.21	0.02, 2.23	0.20

β = measure of how strongly each predictor variable influences the dependent variables, OR = odds ratio, 95%CI = confidence Interval of the odds ratio

* significant p-value.

## Discussion

Majority of the caregivers in this study did not demonstrate appropriate health-seeking behaviour for their under-fives with severe malaria. The initial health intervention for their sick children was sought mainly at the PMVs who are not equipped to provide the required care for these children.^8^,^13^ Although the close-to-client phenomenon of the PMVs could be a major factor warranting the first point of visit during illnesses at the community level; however, some poor practices of these PMVs contribute significantly to poor disease outcome.^8^–^12^,^13^

For example, studies have shown that the prescription pattern of antimalarial drugs, especially for paediatric age groups by the PMVs is poor and most times inaccurate.^24^ Etiaba *et al*^12^ recently documented that the first line visit of most caregivers to the PMVs contributed significantly to wrong diagnosis, inability to detect malaria co-morbidities, worsening of malaria morbidities; and perhaps increased mortality. Most importantly all children with severe malaria are to be referred to health facilities that are equipped with the appropriate material and human resources for prompt management of severe malaria.^1^

Lower family social class was observed to affect the appropriateness of HSB in this study significantly. Caregivers, perhaps, would need to consider what they would pay for transportation and hospital charges before embarking on hospital visit for health care services.^14^–^16^ Due to this, the choice for alternative care outside health facilities is high among the lower social class hence the high patronage of the PMVs as the first line healthcare provider as observed in this study.^8^,^13^ The fact that level of education was not significantly associated with HSB suggests that economic power could be a major factor in the decision to visit PMVs as the first health care provider than to go to the health centres/ hospitals which in most cases cost more.

Accessibility, cost, transportation, increased waiting time in hospitals could be influencing the decision to visit PMVs as the first health care provider than to go to the health facilities.^14^–^16^ Mortality from malaria usually occurs within 2 – 3 days following onset of symptoms of severe disease and is more common in the first 2 – 3 years of life.^3^ In this study, younger age (less than 23 months) was four times a contributory factor to mortality from severe malaria; a finding that is in keeping with that observed by Greenwood *et al*^3^ in the Gambian children. This could be attributed to low level of immunity against the *Plasmodium* species in these children.^25^ Other factors could be socio-cultural such as weaning from breast feeding and social neglect of the child due to presence of a new born in the family as family size interval is usually within two years in most African families.^26^

Severe malaria features that had been documented to be significant contributors to mortality include severe anaemia, cerebral malaria, hypoglycemia, and acute kidney injury.^1^–^4^ Cerebral malaria and jaundice were the features of malaria, significantly predicting mortality in this study. While the diagnosis of hypoglycemia and acute kidney injury are mainly laboratory-based; cerebral malaria features (prostrations, altered sensorium, and coma) and jaundice (yellow eyes) on the other hand are symptoms and signs the caregivers can easily recognise.

Of note is that severe anaemia which usually presents as paleness of the conjunctiva, oral mucosa, hands and feet are also easily identified/ recognised by the caregivers and its morbidities and mortality can be prevented by blood transfusion.^4^ Moreover, Imolele *et al*^27^ had already established that severe anaemia is a good predictor of acute kidney injury in children with severe malaria. It could then be inferred from this study that recognition of severe pallor/ anaemia, jaundice and features of cerebral malaria by caregivers can be life-saving. Therefore, malaria mortality can be prevented by educating caregivers on early recognition of these signs and seeking for prompt care in health facilities. There is also the need for intensified ACSM scheme for malaria prevention/control which will include the need for early presentation / access to prompt treatment of malaria for all members of the community irrespective of their educational status.

### Limitation of study

This was a hospital-based study hence its findings may have under-estimated the effect of HSB on malaria morbidity and mortality. For example, the children who did not present in hospital were not included in this study and these children would have died following the malaria illness at home. A longitudinal communitybased prospective study is, therefore, suggested to bridge the above limitations.

## Conclusion

This study has demonstrated that HSB is an important determinant of outcome of severe malaria in under-fives. Efforts to improve access and affordability of care in health facilities may improve HSB of caregivers of under-fives.

## References

[1] (2012). World Health Organisation training module on malaria control. Case management guide for tutors.

[2] World Health Organization 2017 Report.

[3] Greenwood BM, Bradley AK, Greenwood AM (1987). Mortality and morbidity from malaria among children in a rural area of the Gambia, West Africa. Trans Roy Soc Trop Med & Hyg.

[4] English M (2000). Life threatening severe malarial anaemia. Trans Roy Soc Trop Med & Hyg.

[5] Maitland K, Levin M, English M, Mithwani S, Peshu N, Marsh K (2003). Severe *P. falciparum* malaria in Kenyan children: evidence for hypovolaemia. Q J Med.

[6] Sarkar J, Murhekar MV, Shah NK, van Hutin Y (2009). Risk factors for malaria deaths in Jalpaiguri district, West Bengal, India: evidence for further action. Malar J.

[7] von Seidlein L, Olaosebikan R, Hendriksen IC (2012). Predicting the clinical outcome of severe falciparum malaria in African children: findings from a large randomised trial. Clin Infect Dis.

[8] Nwaneri DU, Sadoh AE, Ibadin MO (2017). Impact of home-based management on malaria outcome in under-fives presenting in a tertiary health institution in Nigeria. Malaria Journal.

[9] Odey F, Esu E, Effa E, Udoh E, Oduwole O, Chibuzor M (2013). Management of severe malaria in children under 5 years of age in private and public health facilities in Cross River State, southeastern Nigeria: an audit of current practices. Clinical Audit.

[10] Federal Ministry of Health, National Malaria Control Programme (2010). Advocacy, communication, and social mobilisation strategic framework and implementation plan.

[11] Thander MM, Kyaw MP, Jimba M, Yasuoka J (2015). Caregivers' treatment -seeking behaviour for children under age five in malaria-endemic areas of rural Myanmar: a cross-sectional study. Malar J.

[12] Etiaba E, Onwujekwe O, Uzochukwu B, Uguru N, Okoronkwo I, Adjagba A (2015). What co-morbidities do people with malaria have and what are their patterns of health seeking in Nigeria. Nig J Clin Pract.

[13] Eseigbe EE, Anyiam JO, Ogunrinde GO, Wammanda RD, Zoaka HA (2012). Health care seeking behaviour among caregivers of sick children who had cerebral malaria in Northwestern Nigeria. Malar Res Treat.

[14] Agu AP, Nwojiji JO (2005). Childhood malaria: mother's perception and treatment -seeking behaviour in a community in Ebonyi State, South-east Nigeria. J Comm Med & Pri Hlth Care.

[15] Jimoh A, Sofola O, Petu A, Okorosobo T (2007). Quantifying the economic burden of malaria in Nigeria using the willingness to pay approach. Cost Eff Resour Alloc.

[16] Uzochukwu BSC, Onwujekwe OE (2004). Socioeconomic differences and health-seeking behaviour for the diagnosis and treatment of malaria: a case study of four local government areas operating the Bamako initiative programme in south-east Nigeria. Intl J Equity hlth.

[17] Nigeria malaria indicator survey 2015. Final report.

[18] Araoye MO (2003). Research Methodology with Statistics for Health and Social Sciences. Nathadex Ilorin.

[19] Oyedeji OA, Oluwayemi IO, Afolabi AA, Bolaji O, Fadero FF (2010). Severe malaria at a tertiary paediatric emergency unit in south west Nigeria. Res J Med Sci.

[20] Cheesebrough M (2005). Examination of blood for malaria parasite in district laboratory Practice in tropical countries. Part 1, second edition.

[21] Liu L, Johnson HL, Cousens S, Perin J, Scott S, Lawn JE (2012). Global, regional, and national causes of child mortality: An update systematic analysis for 2010 with time trends since 2000. Lancet.

[22] NCHS Clinical growth charts (black and white). CDC Growth Chart.

[23] Olusanya O, Okpere E, Ezimokhai M (1985). The importance of social class in voluntary fertility control in a developing country. W Afr J Med.

[24] Nwaneri DU, Meremikwu MM, Nwaneri AC, Ibadin MO (2013). Effect of health education on knowledge of patent medicine vendors on malaria case management and control in Southern Calabar District, Nigeria. J Med Biomed Res.

[25] Doolan DL, Dobano C, Baird JK (2009). Acquired immunity to malaria. Clin Microbiol Rev.

[26] Chibwana AI, Mathanga DP, Chinkhumba J, Campbell CH (2009). Socio-cultural predictors of health-seeking behaviour for febrile under-five children in Mwanza-Neno district, Malawi. Malar J.

[27] Imolele VE, Nwaneri DU, Omoigberale AI, Ibadin MO (2017). Clinical markers and predictors of acute kidney injury in under-fives with severe malaria in a tertiary health institution in Nigeria. British Journal of Renal Medicine.

